# 504. Latent HHV7 Infection Attenuates Beneficial Host-Microbe Interactions in the Human Gut

**DOI:** 10.1093/ofid/ofac492.560

**Published:** 2022-12-15

**Authors:** Emma Lauder, Jonathan L Golob

**Affiliations:** University of Michigan, Ann Arbor, Michigan; University of Michigan, Ann Arbor, Michigan

## Abstract

**Background:**

Human Herpes Virus 7 (HHV7) infection results in a lifelong latent infection with over 95% of adults seropositive for HHV7. Lymphocytes are the best understood host cell type for latent HHV7, with the gastric mucosa another site of latent infection. *Herpesviridae* latency affects host processes like interferon signaling, antigen presentation, and proliferation—the same host pathways are implicated in the beneficial responses to commensal microbes.

**Methods:**

Adult human colonic organoids with or without latent HHV7 infection were co-cultured with *B. longum* in asymmetric oxygen conditions that allow for live interaction (F1). The transcriptional state after 24h of interactions was determined with RNA-sequencing. The transcriptional state was compared between axenic (no microbes) or after co-culture and with or without latent HHV7.

F1: Establishing oxygen gradients across epithelial monolayers

**Figure:**
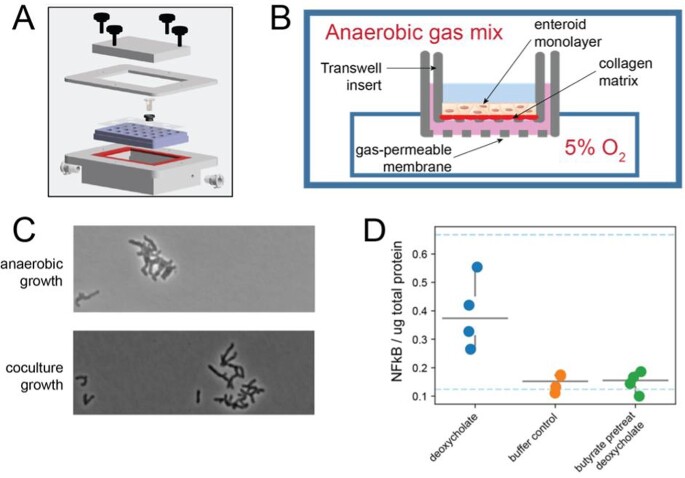

(A) Schematic of oxygen gradient apparatus (B) Closeup view of individual well (C) Growth of the anaerobe B. longum was identical in the apparatus with hypoxic epithelial compared to full anaerobic growth (D) Butyrate administration protects the epithelium from inflammatory effects of deoxycholate.

**Results:**

Screening human intestinal organoids (that lack lymphocytes and are strictly of the endodermal lineage) we have generated evidence that intestinal stem cells (ISC) and IEC can be a host cell for latent HHV7 infection in some but not all people (T1). RNA-sequencing reveals that this is true latency, with ISC and IEC containing HHV7 without transcripts from any of the protein-coding genes in the HHV7 genome. Further we have observed that latent HHV7 is mosaic in the gut, not affecting all of the ISC in an individual (T1).

In organoid-derived human IEC we have noted co-culture with the commensal bacteria *B. longum* suppresses key executors of apoptosis and pyroptosis (F2). These same effects are no longer observed with the *B. longum* is interacting with IEC latently infected with HHV7. A similar pattern was observed with NFkB regulators (F3).

T1: Latent HHV7 is found in some intestinal organoids derived from healthy adults

**Figure:**
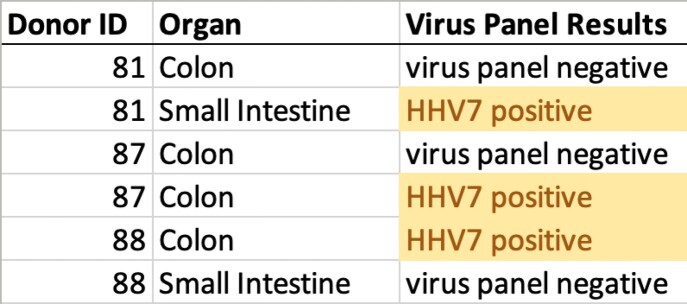

From the University of Michigan Translational Tissue Modeling Laboratory (TTML) organoid registry. Each donor is assigned a unique numerical ID. Viral panel is from a commercial multiplex PCR screen. Notably, donor 87 has colonic organoids with and without latent HHV7.

F2: Co-culture with the commensal microbe B. longum suppresses most apoptosis and pyroptosis executors only in an organoid without latent HHV7 infection.

**Figure:**
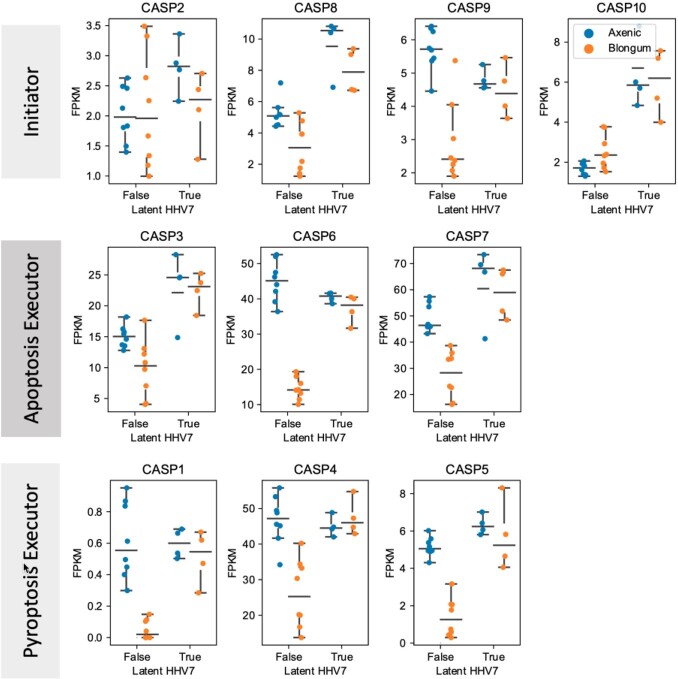

Fragments per kilobase per millions of reads (FPKM) from RNA-sequencing of fully-differentiated organoid-derived human intestinal epithelium after 24 hours of co-culture with B.longum (Orange) or remaining axenic (without microbes; Blue). Results from cell without (left) or with (right) latent HHV7.

F3: Stimulation of the NFkB inhibitor NFKBIA by co-culture with the commensal microbe B. longum is absent in organoids with latent HHV7 infection.

**Figure:**
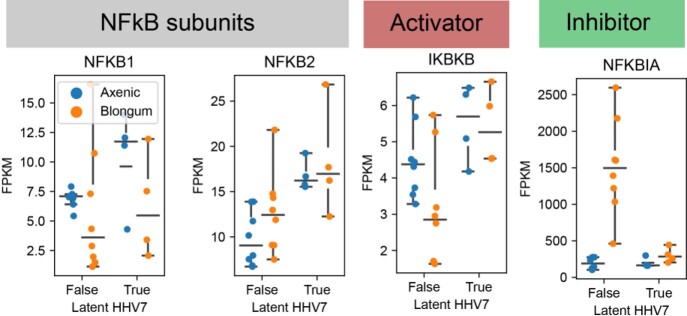

Fragments per kilobase per millions of reads (FPKM) from RNA-sequencing of fully-differentiated organoid-derived human intestinal epithelium after 24 hours of co-culture with B.longum (Orange) or remaining axenic (without microbes; Blue). Results from cell without (left) or with (right) latent HHV7.

**Conclusion:**

Latent HHV7 in the human gut epithelium may attenuate the effect of beneficial microbes like *B. longum* (F4). The evidence for mosaicism of latent HHV7 in the human gut adds a spatial aspect to these attenuating effects that could be relevant for specific disease processes (e.g. skip lesions in Crohn’s disease). Confirmation of these findings, estimating the true prevalence of latent HHV7 in the gut, and identification of the HHV7 latency mechanism will be required to understand the full implications of these findings.

F4: A Model of Latent HHV7 Infection Attenuating Responses to Commensal Microbes.

**Figure:**
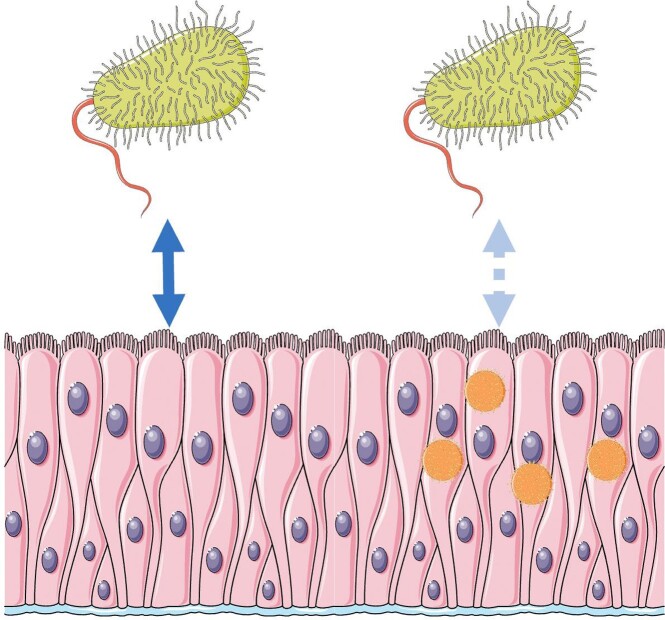

Based on our findings, latent HHV7 infection in the intestinal epithelium may leave the gut mucosa at increased risk for injury by impairing the beneficial response to commensal microbes in a spatially complex manner due to the apparent mosaic nature of latent HHV7 infection in the gut.

**Disclosures:**

**Jonathan L. Golob, MD/PhD**, Loxo Oncology: Employment of Spouse.

